# Self-Rated Health and Subjective Economic Status in Life Satisfaction among Older Chinese Immigrants: A Cross-Sectional Study

**DOI:** 10.3390/healthcare9030342

**Published:** 2021-03-17

**Authors:** Bum Jung Kim, Lin Chen, Ling Xu, Yura Lee

**Affiliations:** 1 Department of Social Welfare, Chung-Ang University, Seoul 06911, Korea; SWL2001@cau.ac.kr; 2 Department of Social Work, Fudan University, Shanghai 200433, China; lin@fudan.edu.cn; 3 School of Social Work, University of Texas at Arlington, Arlington, TX 76019, USA; lingxu@uta.edu; 4 Department of Social Work, Helen Bader School of Social Welfare, University of Wisconsin-Milwaukee, Milwaukee, WI 53201, USA

**Keywords:** self-rated health, subjective economic status, life satisfaction, older Chinese immigrants

## Abstract

This study examines the influence of self-rated health and subjective economic status on the life satisfaction of older Chinese immigrants in the United States. Data were obtained from a cross-sectional survey of 205 older Chinese immigrants aged 66 to 90 years living in Los Angeles and Honolulu. Ordinary Least Squares (OLS) regression analysis was employed to explore the independent effects of self-rated health and subjective economic status. The results demonstrated that self-rated health and subjective economic status were positively associated with life satisfaction. This cross-sectional study provides empirical evidence that self-rated health and subjective economic status are directly associated with subjective life satisfaction among older Chinese immigrants.

## 1. Introduction

Life satisfaction refers to a cognitive evaluation of people’s entire lives, capturing their contentment based on individual, multidimensional criteria [1,2,3,4]. In the literature, life satisfaction is closely associated with the quality of life and successful aging among older adults worldwide [1,3,5,6]. However, few studies have focused on the life satisfaction of older immigrants in the U.S. Immigrants in the United States tend to report lower life satisfaction than non-immigrants [7]. However, Calvo et al. [2] investigated older Hispanic immigrants in the United States and, in contrast, revealed that even with the least socioeconomic resources, they were the most satisfied with their lives among all age groups. Given this unprecedented rise in the number of older immigrants in the United States, this paradoxical finding stresses the need to understand the complex life satisfaction of these older adults [8].

### 1.1. Older Chinese Immigrants

After Hispanic Americans, Asian Americans are the second-largest immigrant group in the United States, and are projected to account for approximately 10% of the country’s population by 2060 [8]. Among all Asian Americans, Chinese Americans are the largest and fastest-growing immigrant group in the nation, accounting for nearly 70% of the entire Asian American population in 2013 [9,10,11]. Although one extensive survey in California showed that the immigrant status of older Chinese immigrant Americans was not likely to cause difficulties, they had poor general English proficiency and were more likely to be uninsured compared with other groups of older Asian Americans [12]. Due to their sizeable population, rapid growth rate, and poor adjustment in their host country, it is worthwhile to examine the life satisfaction of older Chinese immigrants.

Asian immigrants—especially older ones—face various challenges in terms of physical and mental health, language, acculturation, and income [13,14,15]. Moreover, life satisfaction entails considerations of self-rated health and subjective economic status [3,16]. When considering immigrants’ self-rated health and subjective economic status, scholars have often utilized non-Hispanic Caucasians as a reference group [13], which may disguise finely nuanced understandings of minority immigrants’ health and socioeconomic status. Because of their unique culture, traditions, and values, older Chinese immigrants may have perceptions of health and economic status different from those of the general population; furthermore, their views may have distinct influences on their life satisfaction. Thus, this study explores the relationships between self-rated health, subjective economic status, and older Chinese immigrants’ life satisfaction in the United States.

### 1.2. Self-Rated Health and Life Satisfaction

Self-rated health is a powerful predictor of mortality, life satisfaction, and general health [17]. This concept refers to people’s understanding of their own health status and variations that exist among different ethnicities [15,17,18,19]. One study confirmed that ethnic variations significantly affect self-rated health and mortality among different immigrant populations [18]; however, that study failed to detect a specific pattern for Asian immigrants, possibly because it included too many Asian minority subgroups, which may have confounded the outcomes related to ethnicity. Zadjacova and Woo [18] revealed that the notion of self-rated health can be less discriminating when compared among ethnic minority older adults in contrast to non-Hispanic Caucasians, as ethnic minority older adults can be more forgiving of adversities, and hence, report greater life satisfaction.

Different ethnic minority immigrants face distinct obstacles when rating their own health status. For example, despite having access to language-compatible health services, poor spoken English proficiency and inadequate print health literacy remain significant barriers for older Chinese immigrants’ self-rated health [15]. How self-rated health influences older Chinese immigrants’ life satisfaction warrants closer examination.

### 1.3. Subjective Economic Status and Life Satisfaction

Intuitively, people with higher incomes, and therefore, better subjective economic status, are likely to be more satisfied with their lives [4,20,21] since a higher income suggests that a person has more resources, improved subjective economic status, and better life satisfaction [21,22]. Most cross-sectional studies on the relationship between subjective economic status and life satisfaction have shown a robust correlation between these factors, confirming that older adults’ feelings of financial security influence their life satisfaction [3,22]. This may be because older adults focus more on their personal goals than on external resources [5,23], and are likely to adjust their goals according to their current financial resources [22].

From a longitudinal perspective, however, the effects of subjective economic status on life satisfaction are inconsistent throughout an individuals’ life course. Becchetti et al. [24] reported only a small variation in life satisfaction when people’s evaluations of their economic status indicated an improvement over time in the UK [24,25]. In an earlier study, people in the United States exhibited no significant changes in life satisfaction associated with increases or decreases in economic status [20]. Cheung and Lucas [3] further explored the longitudinal effects of subjective economic status associated with life satisfaction over the course of a lifetime. They found that, compared with their midlife counterparts, German and British older adults’ life satisfaction changed less due to shifts in subjective economic status. In contrast, Asian and American older adults’ life satisfaction depends on their economic status. In one study, when Chinese older adults faced economic strain, they reported significantly lower life satisfaction [26,27]. Therefore, the effect of subjective economic status on the life satisfaction of older adults of different ethnicities appears to vary. Since older immigrants may have endured more stress and have been less economically secure in their host country than their counterparts in their home country, the influence of subjective economic status on their life satisfaction needs further investigation [2]. However, few studies have scrutinized the connection between subjective socioeconomic status and life satisfaction among older immigrants.

## 2. Materials and Methods

### 2.1. Sample

A population-based survey was conducted in Los Angeles, California and in Honolulu, Hawaii in 2014 to examine the relationships among self-rated health, subjective economic status, and life satisfaction among older Chinese immigrants. A convenience sampling method was used to gather data from different settings, including day healthcare centers, Chinese cultural centers, senior apartments, and Chinese temples. The inclusion criteria for the participants were that they should be of Chinese origin, living in Los Angeles or Honolulu, aged 65 or older, and be cognitively able to respond to the survey questionnaire. A total of 245 respondents enrolled in the survey and agreed to take part in in-person interviews. Due to missing values or cognitive impairment, the final sample consisted of 205 older Chinese immigrants (an acceptance rate of 83%). The screening process involved administration of the Short Portable Mental Status Questionnaire (SPMSQ); only participants with a score of 8 or above (intact cognitive functioning) were included. Out of the 40 individuals who were not enrolled, 15 were not included due to not meeting the inclusion criteria and 25 were excluded due to cognitive impairment. Professional bilingual social workers interviewed the participants after completing a training session led by the research team.

The participants were informed about the study’s purpose, their rights as interviewees, and were assured of confidentiality. Five bilingual Chinese social workers were trained for the interview process to complete the participant questionnaires and conduct the face-to-face interviews for 30 to 45 min each. This study was approved by the authors’ affiliate university’s Institutional Review Board. Upon completion of the survey, the participants were awarded 20 USD as compensation for their involvement.

### 2.2. Measures

#### 2.2.1. Quality of Life (Subjective Index of Life Satisfaction)

The Campbell quality-of-life index is a standardized survey instrument [28]. The Chinese version for this study was developed using back translation and coordination techniques. The Chinese questionnaire was developed by two bilingual Chinese-American translators with qualifications and experience in social work. The first translator independently translated the questionnaire into Chinese, after which the second translator translated the Chinese version into English. The two translators met and compared the English and Chinese versions of the questionnaire to correct any discrepancies. Itemized analysis was performed to determine that the translation was linguistically and culturally accurate and appropriate. This scale is culturally acceptable and its validity has been established in previous studies carried out among older Chinese individuals [29].

The quality-of-life index contains eight semantic differential scale items: (a) empty or full, (b) useless or worthwhile, (c) lonely or friendly, (d) miserable or enjoyable, (e) boring or interesting, (f) discouraged or hopeful, (g) disappointed or rewarding, and (h) not giving me many opportunities or not giving me the best. The respondents selected and marked the component within each item with an X to indicate their current feelings about life [12]. A high score signaled a high-level quality of life, within a possible score range of 0–56. Campbell et al. [28] reported a reliability coefficient of 0.89 for the quality-of-life index. In this study, Cronbach’s alpha for the index was 0.94.

#### 2.2.2. Self-Rated Health

Self-rated health or personally perceived physical health was the main independent variable in this study. This was assessed using a global question: “How is your health?” There were five possible responses to this question: very poor, poor, neutral, good, or very good.

#### 2.2.3. Subjective Economic Status

We measured subjective economic status with the question: “How would you rate your subjective economic status?” The responses were based on a five-point Likert scale as follows: very low, low, neutral, adequate, or high. This study used the specific question because it has already been used in other studies [5,10] on older Asian immigrants and has been validated and culturally accepted.

#### 2.2.4. Background Information

Demographic information included age (in years), gender (0 = female, 1 = male), marital status (0 = single, 1 = married), individual income, and highest level of education.

### 2.3. Analysis

Descriptive statistics were used to describe the characteristics of the main study variables. Bivariate correlations were employed to check for multicollinearity and to demonstrate the relationships between each independent and dependent variable. Finally, an Ordinary Least Squares (OLS) regression analysis was used to investigate the relative importance of the various independent variables concerning the life satisfaction of older Chinese immigrants using statistical software (STATA version 13) (STATA, city: College Station, TX, USA).

## 3. Results

### 3.1. Descriptive Statistics

Table 1 presents the participants’ descriptive information. The mean age of the sample population was 75.79 (standard deviation (*SD* = ±6.73) with a range of 66–90. Approximately 34% of the respondents were male and 56% of them were married. Their average monthly individual income was 1480 USD (*SD* = ±463.05) and the majority (51.2%) had a high school education, followed by 28.78% with a college education or higher. Concerning self-rated health, the average score for self-rated health was 3.51 (*SD* = ±1.18) and the average score for subjective economic status was 3.58 (*SD* = ±0.60). Lastly, the average score for life satisfaction was 32.52 (*SD* = ±1.20).

### 3.2. Bivariate Analysis

Table 2 summarizes the outcomes of the bivariate analysis among the study variables. Most variables were correlated with life satisfaction, which suggests that a higher level of life satisfaction was observed among those who were younger (r = −0.51, *p* < 0.001), male (r = 0.16, *p* < 0.05), married (r = 0.18, *p* < 0.01), had a higher level of education (r = 0.27, *p* < 0.01), had a higher individual income (r = 0.20, *p* < 0.01), had a high level of self-rated health status (r = 0.57, *p* < 0.001), and had a higher level of subjective economic status (r = 0.44, *p* < 0.001). Interestingly, self-rated health and subjective economic status were negatively associated with education and income, and their relationships were statistically significant. These results do not coincide with our knowledge about the relationship between subjective health and socioeconomic status. In order to understand this unique connection, we need to know the cultural circumstances of older Chinese immigrants. It is usually the case that self-rated health and subjective economic status are positively associated with education/income. Older Chinese immigrants with high levels of education and income have a strong interest and high expectations surrounding health and economic conditions in their later lives. However, as they grow older, they experience more chronic diseases with severe health deterioration, as well as reduced incomes. In other words, their lives do not meet their expectations, resulting in a huge gap. This leads to a lower evaluation of self-rated health and subjective economic status; therefore, older Chinese immigrants with higher education and higher incomes are less likely to experience lower levels of self-rated health status and subjective economic status.

### 3.3. Regression

Table 3 summarizes the findings of Ordinary Least Squares (OLS) regression models. Level 1 includes background information on the participants, Level 2 contains details on their self-rated health, and Level 3 pertains to subjective economic status. In the first model, the demographic variables were entered, which explains 23% of the total variance. Age (β = −0.44, *p* < 0.001) and education level (β = 0.21, *p* < 0.01) significantly predicted life satisfaction. Younger individuals with higher levels of education were likely to have greater life satisfaction levels. Model 2 explains 36% of the variance (F = 39.63, *p* < 0.01). This outcome revealed that, after controlling for socio-demographic characteristics, self-rated health status (β = 0.42, *p* < 0.001) was positively associated with a high level of life satisfaction. Model 3 explains an additional 3% of the variance (F = 46.47, *p* < 0.01). Being younger (β = −0.25, *p* < 0.001), being married (β = 0.11, *p* < 0.05), having a high level of education (β = 0.13, *p* < 0.01), and having a high level of self-rated health status (β = 0.38, *p* < 0.001) were associated with a deeper level of life satisfaction. Interestingly, having a high subjective economic status (β = 0.16, *p* < 0.001) was positively associated with a high level of life satisfaction, whereas income level (which often forms part of the definition of economic status) was not associated with a high level of life satisfaction.

## 4. Discussion

The results imply significant, positive relationships among self-rated health, subjective economic status, and life satisfaction. More specifically, older Chinese immigrants with a very good level of self-rated health and a high level of subjective economic status were more likely to show a high level of life satisfaction.

Participants with excellent self-rated health status had higher levels of life satisfaction than their counterparts with a lower self-rated health status. These results are consistent with previous studies that have examined the relationship between health status and life satisfaction among older Asian populations. A study of older Chinese people in Hong Kong also revealed that self-rated health, along with socio-economic status, had a critical effect on their life satisfaction. In another study, self-rated health was positively and significantly associated with life satisfaction among older adults in mainland China. These results are similar to those reported for other older immigrant groups in the United States [30,31,32,33,34].

Based on the study findings, which highlight the importance of the relationship between self-rated health and life satisfaction among older Chinese in the sample, various health promotion programs at the community level should be investigated, developed, and tested. Furthermore, community leaders and social workers should increase funding for existing programs and increase public awareness campaigns to emphasize the importance of promoting health among older Chinese immigrants. Considering that they tend to live alone or away from their families, social workers should play an active role in encouraging them to participate in community-based health care services to enhance their physical function and mobility. Finally, it is vital to provide sufficient user-friendly, health-related information and services to enable professional bilingual staff to offer culturally appropriate and effective services.

This study showed that income level was not significantly related to the level of life satisfaction, while subjective economic status was significantly and positively associated with a higher level of life satisfaction. According to Wang et al. [33], a considerable number of older Chinese immigrants rely heavily on financial support from their families and the government due to poverty later in life. Traditionally, based on the understanding derived from Confucianism and filial piety, families (including children and other relatives) have been the main source of economic support for older Chinese people. However, this situation has shifted, as most older Chinese want to live independently and not place a financial burden on their children, who may be experiencing financial difficulties [33]. This could be why many older Chinese immigrants living in poverty, and lacking financial resources, look for jobs with higher incomes to become more self-reliant.

Furthermore, older Chinese immigrants find it difficult to find suitable jobs later in life [12,35]. There are several possible reasons for this. First, throughout the history of immigration, numerous older Chinese immigrants have moved to the United States in their earlier adult lives to pursue the “American dream” and a better life, and to gain education for their children in an advanced academic environment. Following their migration to the United States, they sought skilled jobs with higher pay; however, their generally poor English language proficiency resulted in employment in labor-intensive/low-skilled industries or self-employment in small businesses. As with other immigrants, having to work long hours and often having multiple jobs makes it difficult to participate in English learning programs and to adapt to the culture and values of the mainstream social system in the United States [33]. In addition, the focus on their children’s higher education leads to decreased savings, and many older Chinese immigrants have had to find work to improve their economic situation later in life.

Economic status had a greater influence on their life satisfaction. Those with a higher subjective economic status at the time of the study tended to report greater levels of life satisfaction. This finding is consistent with preceding studies that suggest a higher subjective economic status has a positive influence on life satisfaction. Research on older Chinese individuals in mainland China demonstrated that subjective economic status and life satisfaction had a significant, positive relationship. Moreover, in most studies on older immigrants in the United States, subjective economic status had a significant effect on life satisfaction [12,30,35].

The results of this study indicated that age, education, and marital status had a significant influence on the participants’ life satisfaction. Older Chinese immigrants who were relatively younger, married, and had higher levels of education were more likely to have greater levels of life satisfaction.

This study faced several limitations. First, due to the cross-sectional design, no causal relationship could be detected between self-rated health, subjective economic status, and life satisfaction. Second, the study used non-probability convenience sampling, primarily in public places with a small number of participants. The use of convenience sampling may have introduced selection bias through oversampling people within average or lower levels of income categories or less healthy individuals. Given the nature of the sample, the findings should be viewed with caution, and further investigations with other samples or longitudinal assessments are required. Additionally, future studies should include more participants in each study site in order to distinguish among group differences. Third, the measurement of subjective economic status in the analysis was linear, and did not consider varying types of economic security or to what extent varying levels of subjective economic status affected the outcome variable; more detailed investigation is needed in this regard. In addition, the measurement of marital status was linear and did not consider specific details of unmarried (single, divorced, bereaved) individuals; as such, further examination is needed. Future studies should consider employing more frequently used and validated measures to gauge subjective economic status. Lastly, the study did not encompass other significant variables that could affect life satisfaction (e.g., length of stay in the host country, social engagement, social support, and employment status) because the original data did not contain variables. Thus, this should be explored more in depth.

## 5. Conclusions

Despite its limitations, this study clearly demonstrated that self-rated health is associated with life satisfaction among older Chinese immigrants in the United States. The results imply that older Chinese immigrants’ subjective economic status is significantly associated with life satisfaction. These findings emphasize the importance of economic and health status indicators for the well-being of older immigration populations.

## Figures and Tables

**Table 1:** Descriptive information (*n* = 205).

Variable	*n*	%
Age (years)		data
Range	66–90	
Mean (*SD*)	75.79 (6.73)	
Gender		
Female	135	63.29
Male	70	34.71
Marital status		
Not married	91	43.37
Married	114	56.63
Individual income		
Range	0–3850	
Mean (*SD*)	1480.87 (463.05)	
Education		
Elementary and below	29	14.1
Middle	12	5.85
High school	105	51.22
College and above	59	28.78
Self-rated health	1–4	
Range	3.51 (1.18)	
Mean (*SD*)		
Subjective economic status	1–5	
Range	3.58 (.60)	
Mean (*SD*)		
Life satisfaction	16.03–48.65	
Range	32.52 (3.20)	
Mean (*SD*)		

Note. *SD* = standard deviation.

**Table 2:** Correlations among the study variables (r-scores, *n* = 205).

Variables	1	2	3	4	5	6	7
1. Age	–						
2. Gender	−0.06						
3. Marital status	−0.16 *	0.30 **					
4. Education	−0.13	0.21 **	−0.14 *				
5. Income	−0.21 **	0.25 **	0.13 *	0.18 **			
6. Self-rated health Status	0.43 ***	−0.38 ***	−0.09	−0.29 ***	−0.22 **		
7. Subjective economic status	−0.40 **	0.25 **	0.21 **	−0.19 **	−0.40 **	0.40 **	
8. Life satisfaction	−0.51 ***	0.16 *	0.18 **	0.27 **	0.20 **	0.57 ***	0.44 ***

Note: * *p* < 0.05. ** *p* < 0.01. *** *p* < 0.001. Gender: Female = 0, Male = 1, Marital status: Single = 0, Married = 1.

**Table 3:** Ordinary Least Squares (OLS) regression models of life satisfaction (*n* = 205).

Variables	Model 1		Model 2		Model 3	
	Beta	*t*-Value	Beta	*t*-Value	Beta	*t*-Value
Age	−0.45	−9.10 ***	−0.29	−5.33 ***	−0.25	−4.40 ***
Male	0.04	0.59	−0.09	−01.51	−010	−1.72
Married	0.11	1.91	0.13	2.41 *	0.11	2.11 *
Education	0.21	4.84 **	0.14	3.49 **	0.13	3.16 **
Income	0.04	1.67	0.02	1.28	0.01	−095
Self-rated health			0.42	6.05 ***	0.38	5.62 ***
Subjective economic status					0.16	3.25 ***
R^2^	0.23		0.34		0.37	
R^2^ change			0.11 ***		0.02 **	
Adjusted R^2^	0.21		0.36		0.39	
F	26.51 **		39.63 ***		46.47 **	

Note. * *p* < 0.05. ** *p* < 0.01. *** *p* < 0.001.

## Data Availability

The data for this study are available upon request (originally collected as raw data).
